# Relapsed Acute Lymphoblastic Leukemia

**DOI:** 10.1007/s12098-023-04635-4

**Published:** 2023-06-21

**Authors:** Jasmeet Sidhu, Manash Pratim Gogoi, Shekhar Krishnan, Vaskar Saha

**Affiliations:** 1 Department of Pediatric Hematology and Oncology, Tata Medical Center, Kolkata, 700160 India; 2 Tata Translational Cancer Research Center, Tata Medical Center, Kolkata, 700160 India; 3 University Children’s Hospital, Zurich, 8008 Switzerland; 4 Division of Cancer Sciences, School of Medical Sciences, Faculty of Biology, Medicine and Health, University of Manchester, Manchester, M20 4BX UK

**Keywords:** Relapse, Childhood ALL, Chemotherapy, Transplant

## Abstract

Outcomes for children with acute lymphoblastic leukemia (ALL) have improved worldwide to >85%. For those who relapse, outcomes have remained static at ~50% making relapsed acute lymphoblastic leukemia one of the leading causes of death in childhood cancers. Those relapsing within 18 mo in the bone marrow have a particularly dismal outcome. The mainstay of treatment is chemotherapy, local radiotherapy with or without hematopoietic stem cell transplantation (HSCT). Improved biological understanding of mechanisms of relapse and drug resistance, use of innovative strategies to identify the most effective and least toxic treatment regimens and global partnerships are needed to improve outcomes in these patients. Over the last decade, new therapeutic options and strategies have been developed for relapsed ALL including immunotherapies and cellular therapies. It is imperative to understand how and when to use these newer approaches in relapsed ALL. Increasingly, integrated precision oncology strategies are being used to individualize treatment of patients with relapsed ALL, especially in patients with poor response disease.

## Introduction

Outcomes for childhood acute lymphoblastic leukemia (ALL) have improved to >85% worldwide with risk-stratified response-based chemotherapy regimens. However, irrespective of treatment protocols used, 10–15% of patients relapse, making relapsed ALL one of the leading causes of death in childhood cancer. Over the last decade, newer therapies have become available—immunotherapies, chimeric antigen receptor T-cells (CAR-Ts) and targeted agents in biologically relevant subsets which have increased the hope for improving cure rates. Here, authors review the current management strategies and newer approaches for treatment of relapsed childhood ALL.

## Biology of Relapsed ALL

**Fig. 1:**
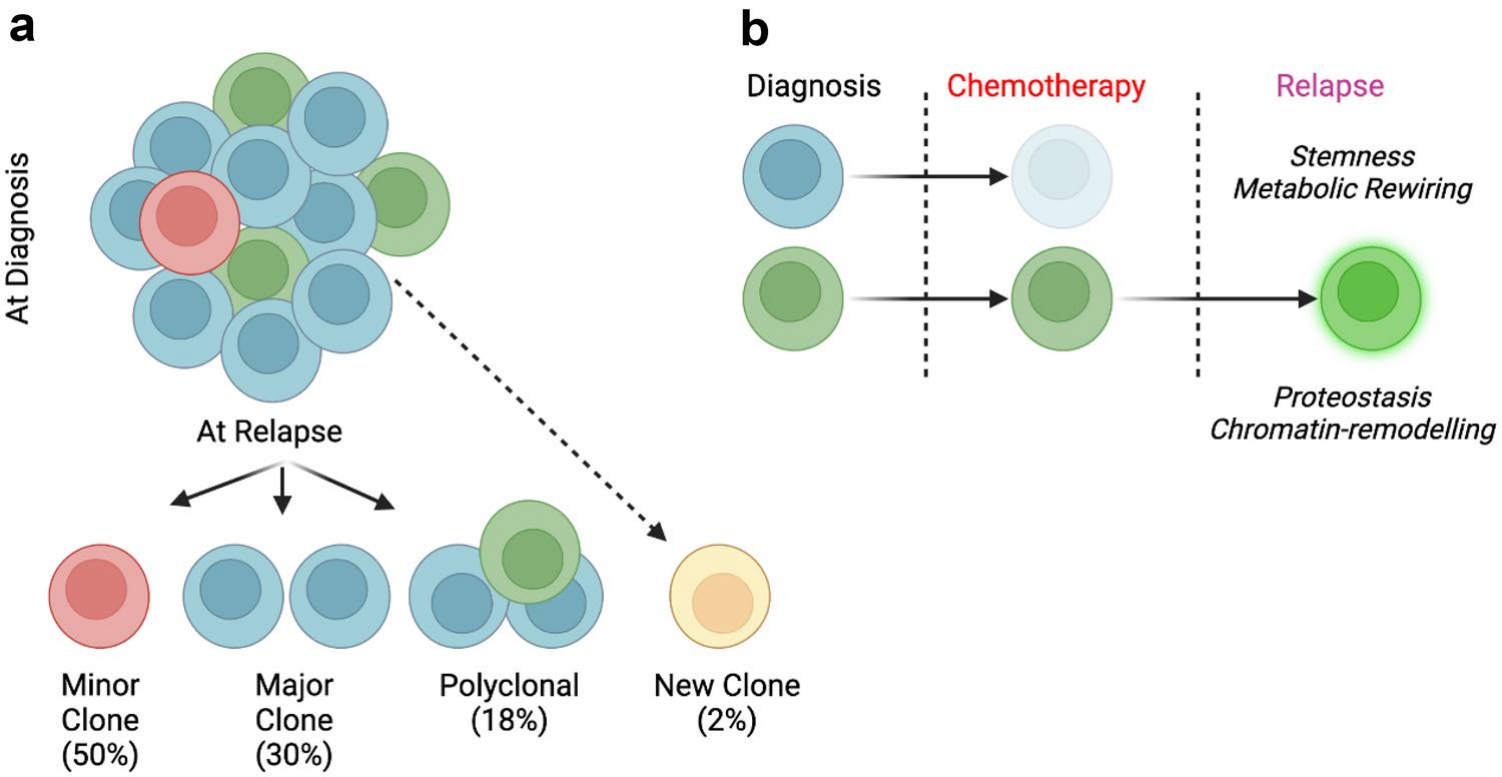
(**a**) Evolution of subclones at relapse (**b**) Mechanisms of drug tolerance of relapse-fated clones. Adapted from [3, 4]

ALL has considerable genetic heterogeneity, both at chromosomal and at single gene level. Karyotype, copy number alterations and single gene mutations have prognostic value in relapsed ALL [1]. High risk cytogenetics, deletions of *NR3C1* and *BTG1* and mutations of *TP53* and *NRAS* are associated with treatment failures [2]. ALL is a polyclonal disease and recurrence is usually due to expansion of subclones present at diagnosis. In 50% of cases, a minor clone present at diagnosis is dominant at relapse; in around a third, the major clone is present and in about 20%, relapse is polyclonal (Fig. 1a) [3]. Subclonal mutations associated with relapse include those affecting *CREBBP*, *NOTCH1* and Ras family genes [2]. Rarely, disease occurs due to additional mutations that arise, possibly due to therapy, in ancestral clones. These may include mutations in *NCOR2*, *USHA2*, *NT5C2* [3, 5] and *TP53* [6]. Patient-derived xenograft models suggest that relapse-fated clones are drug tolerant and are enriched in chromatin re-modelling, mitochondrial metabolism, proteostasis and have a “stem-cell” like profile (Fig. 1b) [4].

## Risk Stratification at First Relapse

The duration of first complete remission (CR1), site of relapse and immunophenotype are used for stratification in relapsed ALL [7–9]. ALL-REZ-BFM 2002 [10], ALLR3 [9], and InPOG-ALL-R1 [11] protocols classified time to relapse as very early (within 18 mo of first diagnosis), early (after 18 mo of first diagnosis but within 6 mo after end of therapy), and late (more than 6 mo after end of therapy). The Berlin Frankfurt Münster (BFM) group initially classified late extramedullary (EM) relapses as S1; very early/early EM relapses, early and late combined and late medullary (all non-T) as S2; early isolated medullary non-T relapses as S3; and very early medullary or combined (non-T) and any T-ALL relapse with medullary involvement as S4. Subsequently ALLR3 classified S1 as standard risk (SR); S2 as intermediate risk (IR) and S3-S4 as high risk relapses. The IntReALL group (NCT01802814) simplified these further (Table 1). The Children’s Oncology group (COG) used a similar risk stratification but defined early relapse as marrow relapse <36 mo from diagnosis or isolated extramedullary relapse <18 mo from diagnosis [12]. Additionally, factors like older age (>10 y), central nervous system (CNS) disease, male gender and high-risk genetics have also been shown to influence outcomes in relapsed ALL [1, 2, 13].

**Table 1:** Risk stratification, relapsed childhood acute lymphoblastic leukemia (ALL)

**COG**	
Low	Late B-ALL marrow, end-block 1 MRD <0.1%
	Late IEM, end-block 1 MRD <0.1%
Intermediate	Late B-ALL marrow, end-block 1 MRD ≥0.1%
	Late IEM, end-block 1 MRD ≥0.1%
High	Early B-ALL marrow
	Early IEM
	T-ALL relapse, any site and timing
**BFM Group, ALL R3**	
Low (S1)	Late IEM relapses
Intermediate (S2)	Very early and early IEM relapses
	Late B-ALL isolated marrow
	Early/Late B-ALL combined
High (S3 and S4)	Very early and early B-ALL marrow
	Very early B-ALL combined
	T-ALL marrow at any time
**IntReALL 2010**	
SR	Late isolated or Late/early combined B-ALL marrow
	Any late/early isolated extramedullary relapse
HR	Early/Very early isolated marrow relapse
	Very early isolated/combined extramedullary relapse
**InPOG-ALL-R1**	
SR	Late isolated extramedullary;
	Late B-ALL medullary with EOI MRD <0.01%;
	Early isolated testes relapsed B-ALL with non-high risk genetics
	Age <15 y at diagnosis
HR	All very early, all medullary T-ALL, early marrow, and non-testes extramedullary
	Late B-ALL medullary relapses with end of induction MRD ≥0.01%
	Mixed Phenotype Ambiguous Lineage
	High risk genetics
	Age ≥15 y

*BFM* Berlin-Frankfurt-Münster, *COG* Children’s Oncology Group, *EOI MRD* End of induction minimal residual disease, *HR* High risk, *IEM* Isolated extramedullary, *InPOG* Indian Pediatric Oncology Group, *IntReALL* International relapsed ALL study group, *MRD* Minimal residual disease, *SR* Standard risk

## Treatment of Relapse – The Chemotherapy Era

The ALL-REZ-BFM 85 trial identified duration of CR1, site of relapse and immunophenotype as predictive of outcome after relapse [14]. Patients with very early and early relapses do poorly as compared to those with late relapses [8, 9]. COG, North America study analyzed 1961 patients who were treated for relapse with different COG protocols between 1988–2002 [13]. Patients relapsing within 18 mo of initial diagnosis had worse outcomes [5-y overall survival (OS): isolated medullary relapse 11%, combined medullary relapse 11.5%, isolated CNS (iCNS) relapse 43.5%] as compared to late relapses (5-y OS: isolated medullary relapse 43.5%, combined medullary relapse 60%, iCNS relapse 78%). Isolated medullary relapses have worst outcomes, followed by combined medullary and extramedullary relapses, followed by isolated extramedullary relapses [8, 13]. Patients with minimal residual disease (MRD) levels <10^–3^ at the end of induction treatment had an event-free survival (EFS) of 86% compared to 0% for those with MRD of ≥10^–3^ at end of induction [15]. This was confirmed in a larger cohort of patients [16]. Table 2 provides a synopsis of recently completed clinical trials for first ALL relapse**.**

**Table 2:** Phase 3 Clinical Trials for relapsed acute lymphoblastic leukemia (ALL)

Group	Years	Age (years)	Type of relapse	No. of patients	EFS/PFS	OS	Comments
ALL R3	2003–2009	1–18	First relapse	239 (216 randomized)	3-y Mitoxantrone arm: 64.6%; Idarubicin arm: 35.9%	3-y Mitoxantrone arm: 69%; Idarubicin arm: 45.2%	Mitoxantrone arm more effective than idarubicin arm
ALL-REZ-BFM 2002	2003–2012	1–18	First relapse	538 (420 randomized)	5-y Prot II-IDA arm: 60%; R arm: 53%	5-y Prot II-IDA arm: 69%; R arm: 63%	Post-induction therapy with Protocol II-IDA was associated with fewer subsequent relapses compared to R-courses
DCOG Rel-ALL 98	1999–2006	1–18	Early and late	158 (99 reported)	5-y Early: 12%; Late: 35%		Early relapses have poorer outcome
NOPHO (multiple protocols)	1992–2011	1–14.9	First relapse	516	5-y: 44%	5-y 51.5%	Improved outcomes over time mainly due to more late relapses over the years
COG AALL0433	2007–2013	1–30	Late medullary or very early iCNS relapsed B-ALL	275 (271 eligible)	3-y late medullary 77.5%; early iCNS 41.4%	3-y late medullary 81.5%; early iCNS 51.7%	HSCT did not improve OS in late medullary relapses. Early iCNS relapses do poorly
COG AALL1331	2014–2019	1–30	IR/HR	220 (208 randomized)	59.3% (blinatumomab arm)	79.4% (blinatumomab arm)	Blinatumomab superior to standard chemotherapy as post-reinduction consolidation in IR/HR relapses
NCT02435849	2015–2017	3–12	CD19+ relapsed or refractory B-cell ALL	75	1 y: 50%	1 y: 76%	Durable remission with single infusion of tisagenlecleucel (CTL019)

*BFM* Berlin-Frankfurt-Münster, *COG* Children’s Oncology Group, *DCOG* Dutch Childhood Oncology Group, *EFS* Event-free survival, *HR* High risk, *HSCT* Hematopoietic stem/progenitor cell transplantation, *iCNS* Isolated central nervous system, *IDA* Idarubicin, *IR* Intermediate risk, *NOPHO* Nordic Society for Pediatric Hematology and Oncology, *OS* Overall survival, *PFS* Progression-free survival, *Prot* Protocol

The ALL-REZ-BFM 2002 and ALLR3 trials stratified late B-cell precursor ALL (BCP-ALL) relapses for treatment with chemotherapy *vs.* allogeneic hematopoietic stem cell transplantation (HSCT) based on MRD levels at the end of induction (EOI MRD). The BFM used a non-anthracycline based induction protocol and an EOI MRD cut-off of 10^–3^. ALLR3 used an anthracycline-based induction protocol and an EOI MRD cut-off of 10^–4^. In both protocols, patients with late isolated extramedullary relapse were not offered HSCT, while patients with early and very early medullary relapses (isolated and combined) were eligible for HSCT. All T-ALL patients with medullary relapse (isolated or combined) were eligible for HSCT. Results of both trials confirmed cure without HSCT (EFS rates, ~72%) in BCP-ALL patients with late bone marrow relapse who achieve low EOI MRD (<10^–4^) [2, 17]. The BFM observed comparable outcomes between patients with EOI MRD <10^–3^ and EOI MRD 10^–3^ to 10^–4^; however, unlike in ALLR3, these patients additionally received cranial irradiation in the BFM protocol. In patients with late BCP-ALL medullary relapse and high EOI MRD, outcomes were better with HSCT [17]. Combined data from both trials showed continuing poor outcome of early/very early BCP-ALL medullary relapse (isolated or combined) and T-ALL medullary relapse (22.6% and 26.2% respectively). In this high risk group, about a third failed to achieve remission and only half reached the time point for HSCT. ALLR3 examined idarubicin *vs.* mitoxantrone in induction. Surprisingly, mitoxantrone showed a significant survival benefit over idarubicin (*P* = 0.0004) [9]. The COG AALL0433 transplanted all BCP-ALL late medullary relapses where a matched sibling stem cell donor was available. Patients with EOI MRD <10^–3^ had a 3-y EFS of 84.9% [18].

**Fig. 2:**
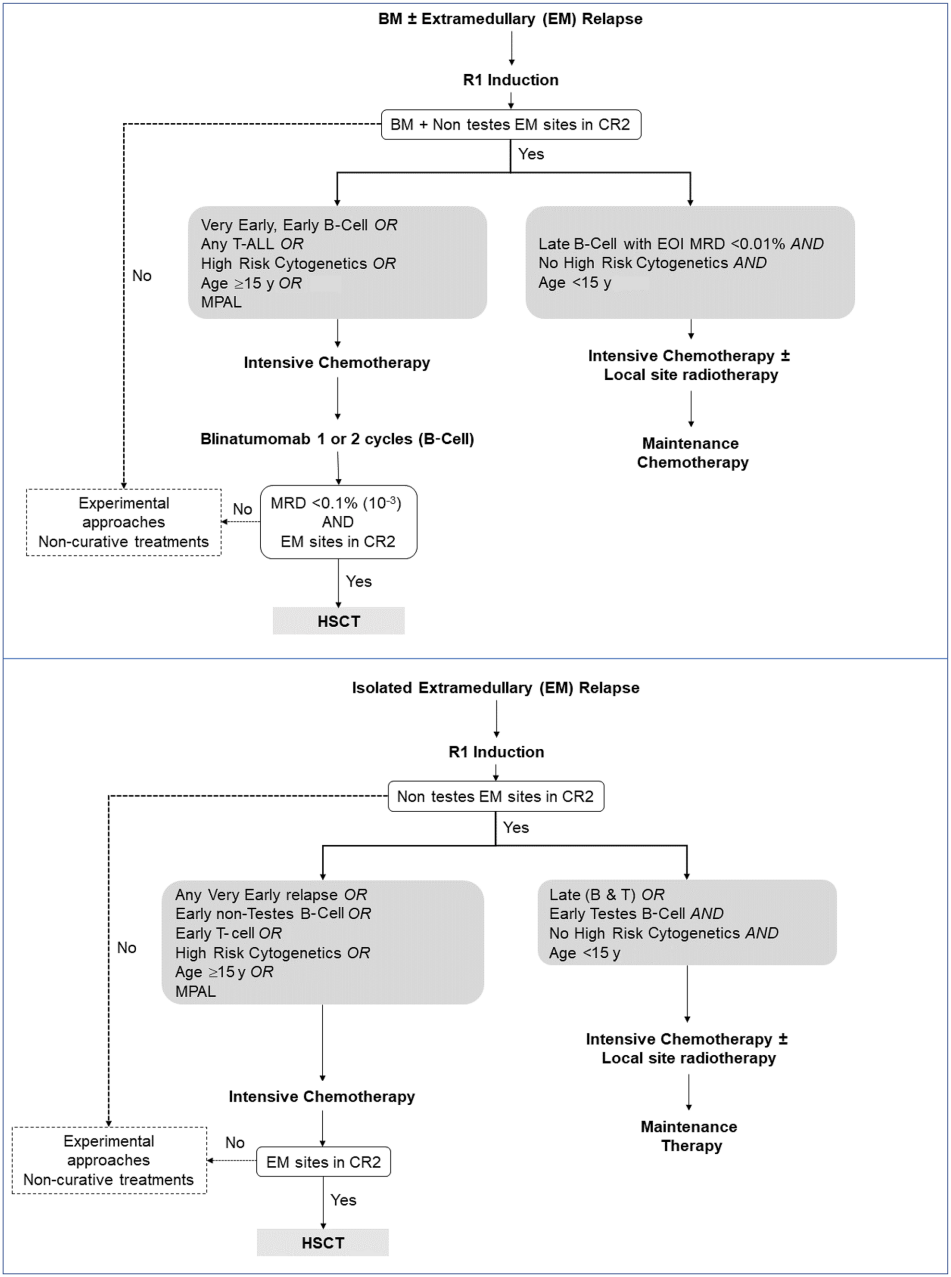
Treatment schema of the InPOG-ALL-R1 treatment protocol for patients with untreated first relapse of acute lymphoblastic leukemia (InPOG-ALL-19–02-ALL R1; Clinical Trials Registry-India CTRI/2019/10/021758). Upper panel: Patients with medullary (bone marrow) ALL relapse, either isolated or combined (marrow relapse combined with relapse at extramedullary sites); Lower panel: Patients with isolated extramedullary relapse. High risk cytogenetics include Philadelphia chromosome-positive ALL, other ABL-class ALL, KMT2A-rearranged ALL, ALL with intrachromosomal amplification of chromosome 21 (iAMP21), TCF3-rearranged ALL, ALL with hypodiploidy (modal chromosome number <40) and ALL with select gene copy number alterations (such as TP53 deletion). *BCP-ALL* B cell precursor-ALL, *BM* Bone marrow, *CR2* Complete remission second, *EOI MRD* End of induction minimal residual disease, *HSCT* Hematopoietic stem/progenitor cell transplantation, *MPAL* Mixed phenotype acute leukemia

COG AALL07P1 evaluated the addition of bortezomib to a 4-drug induction backbone in patients with high-risk ALL relapse and observed remission rates similar to those reported by the BFM/ALLR3 studies. Of 135 patients treated with bortezomib as part of induction, 68% achieved second remission (CR2) [19]. At authors’ center, they found the ALLR3 protocol to be too intensive for some of their patients. They replaced vincristine in induction with bortezomib and reduced the dose of cytarabine in third block [11]. This protocol has produced outcome results comparable to those reported by BFM and ALLR3 groups, with lower toxicity. The schema for InPOG-ALL-R1 protocol (CTRI/2019/10/021758) is shown in Fig. 2.

Patients with late (isolated or combined) bone marrow relapse (relapse ≥6 mo from end of treatment of first presentation ALL), with EOI MRD <10^–4^ and without other high risk features (age ≥15 y, high risk cytogenetics) are treated with chemotherapy alone. Chemotherapy includes an intensive phase that includes induction, consolidation and intensification treatment blocks, followed by the interim maintenance and maintenance treatment phases. Where involved, extramedullary disease sites are irradiated. Patients with late isolated extramedullary disease (and a subset of boys with early isolated testicular relapse of BCP-ALL) who have no high risk features are similarly treated with chemo-radiotherapy alone. All other patients are treated with intensive chemotherapy followed by HSCT. Where available, patients with marrow relapse of BCP-ALL who require HSCT are administered 1–2 courses of blinatumomab (a CD19-targeting, T-cell engaging antibody) prior to transplant. Patients who do not achieve a second remission (at marrow and/or non-marrow disease sites) have difficult-to-cure relapse and require alternative approaches.

Although somatic genetic alterations have not been usually considered for risk stratification, unfavorable genomic alterations can influence outcome [1, 2]. Combined ALL-REZ-BFM 2002 and ALLR3 data suggests higher rates of induction failure in patients with *TCF3* and *KMT2A* rearrangements, hypodiploidy (<40 chromosomes) and copy number alterations of *IKZF1, BTG and NR3C1* [8]. *TP53* alterations are observed in 10–12% patients at time of relapse and HSCT should be considered in CR2 in these patients irrespective of MRD response [20]. Analysis of late bone marrow relapses in UKALL R3 showed that patients with deletion/mutation of *IKZF1/PAX5/NR3C1/NRAS* had inferior progression-free survival (PFS);(PFS 50%, 10% lower than the overall cohort) [17].

### Role of HSCT

As discussed earlier, both ALL-REZ-BFM 2002 and ALLR3 showed improved outcomes with HSCT in patients with late medullary relapses and high EOI MRD (EOI MRD ≥10^–4^) [10, 17]. Pre-transplant MRD level is prognostic of outcomes [21]. Among patients who do not achieve MRD negativity pre-transplant, those with low MRD (MRD <10^–3^) do better (EFS 39%) than those with high pre-transplant MRD (MRD ≥10^–3^, EFS 18%) [21].

ALL-SCT-BFM-2003 was the first trial comparing outcomes and treatment-related mortality with HLA-matched sibling donor and HLA-matched unrelated donor HSCT and no significant differences in EFS, OS and non-relapse mortality were observed between the two groups or, between peripheral blood stem cell and bone marrow grafts [22]. A recent study showed improved HSCT outcomes regardless of donor type in recent cohorts as compared to older cohorts (sibling, 70% *vs.* 24%; unrelated, 61% *vs.* 37%; and haploidentical, 88% *vs.* 19%) in very-high risk pediatric leukemia patients [23]. This was attributable to lower rates of infection [hazard ratio (HR) = 0.12; *P* = 0.005], regimen-related toxicity (HR = 0.25; *P* = 0.002), and leukemia-related death (HR = 0.40; *P* = 0.01). Notably, higher leukemia-free survival was observed with haploidentical HSCT in this study, related possibly to a lesser toxicity non-total body irradiation (non-TBI) HSCT-conditioning regimen and better post-transplant leukemia control through comprehensive killer immunoglobulin-like receptor typing of haploidentical donors. A randomized study comparing TBI with chemotherapy-conditioning showed poorer survival outcomes with the latter (2-y OS, 91% *vs.* 75%, *P* <0.0001). The 2-y cumulative incidence of relapse and treatment-related mortality were 12% and 2% with TBI conditioning *vs.* 33% and 9% respectively following chemotherapy conditioning [24].

### T-ALL Relapse

Most relapses in T-ALL usually occur early [25], are mostly high risk and require HSCT as definitive treatment. Patients with early T-ALL relapse experience high induction failure (42% *vs.* 11% in late relapse) [8]. Post-HSCT disease-free survival (DFS) and OS was 51.6% and 55.4% in the combined ALL-REZ-BFM 2002 and ALLR3 analysis of T-ALL relapse [8].

Purine analogue, nelarabine, has been shown to be effective in T-ALL. Response rates of more than 50% were seen in first relapse of T-ALL in a COG study, however, 18% patients had grade ≥3 neurotoxicity [26]. Bortezomib is another effective agent for T-ALL (the COG AALL1231 study) [27]. In a phase I dose escalation study (NCT03181126) that included 18 T-ALL patients, remission was achieved in 10 patients (55.6%) with T-ALL treated with the venetoclax-navitoclax combination [28].

### Isolated Extramedullary Relapse

An analysis of over 9000 patients with first relapsed ALL treated in COG trials reported survival at 5 y for patients with very early, early and late isolated CNS (iCNS) relapse as 44%, 68% and 78% respectively, and 14%, 52% and 60% for very early, early and late isolated testicular relapses [13]. Extramedullary relapse is considered a regional manifestation of systemic ALL recurrence [29]. Treatment, thus, involves systemic therapy along with local irradiation (administered at the end of intensive chemotherapy to allow delivery of chemotherapy without interruption). The standard dose for cranial irradiation is 24 Gray (Gy) [30], though lower doses are being considered to minimize long-term toxicity. In one study, 18 Gy was sufficient in early iCNS relapse patients [31]. For very early and early iCNS relapses, both COG AALL0433 and ALLR3 show benefit of HSCT after systemic chemotherapy [7, 30]. For isolated testicular relapse, local therapy in form of orchidectomy of the involved testis (if causing significant discomfort) or bilateral irradiation is given. In COG AALL02P2, only patients with persistent testicular enlargement and biopsy-proven disease were given testicular irradiation [32].

## Newer Therapies

### Immunotherapies

Blinatumomab is a bispecific T-cell engager (BiTE) which binds CD3-positive T-cells to CD19-positive leukemic cells, leading to T-cell activation-mediated leukemic cell kill. In the COG AALL1331 trial, 208 patients (ages 1–30 y, with first intermediate/high risk relapse of BCP-ALL) were randomized post-induction to receive 2 blocks of intensive chemotherapy (Arm A) or 2 cycles of blinatumomab (Arm B) [12]. Randomization was closed early due to improved DFS (59% *vs*. 41%), superior OS (79% *vs.* 59%), lower toxicity, and superior MRD clearance (79% *vs.* 21%) for Arm B relative to Arm A. Cytokine release syndrome, neurotoxicity and infections are associated toxicities.

Inotuzumab ozogamicin (InO) is an anti-CD22 monoclonal antibody conjugated to the cytotoxin calicheamicin. In a study involving 51 children with relapsed/refractory ALL, treatment with InO resulted in CR in 67% patients, 71% of whom achieved MRD negativity [33]. Hepatotoxicity (12%) and infections (22%) were the principal toxicities. Significantly higher rates of hepatic sinusoidal obstruction syndrome (52%) were observed with HSCT in InO-treated patients. The IntReALL 2010 protocol tested use of another CD22-directed antibody, epratuzumab, as a randomized intervention post-induction in standard risk ALL relapses. The results of this randomization have not yet been published.

Daratumumab, a CD38-directed humanized monoclonal antibody, was used in a recent study involving pediatric patients with relapsed/refractory T-ALL. Forty two percent achieved complete remission after 1 cycle of daratumumab in combination with prednisolone, vincristine, asparaginase, and doxorubicin [34]. Long-term studies are needed to establish daratumumab’s role in relapsed T-ALL treatment.

### Cellular Therapies

Autologous or allogeneic T-cells or natural killer (NK) cells can be genetically modified to attack leukemic cells. T-cells transduced with chimeric antigen receptor (CTL019) lentiviral vector (Tisagenlecleucel) were infused in 75 children with relapsed or refractory CD19 + BCP-ALL [35]. The EFS and OS were 73% and 90% respectively, at 6 mo and 50% and 76% at 12 mo. Since then, there has been an increased interest in use of CAR-Ts in relapsed pediatric ALL. Recently, at least two Indian centers have reported the development of CAR-Ts for use in lymphoid malignancies [36, 37]. Cytokine release syndrome and neurotoxicity are the principal toxicities. Exhaustion of CAR-T cells over time is believed to be a significant cause of relapse post CAR-infusion. High costs have limited their wide use. While CAR-T cells have been used primarily for BCP-ALL, recently base editing technology has been used to successfully engineer CAR-T cells for T-cell ALL [38]. NK cells can also be modified ex-vivo to target leukemic cells through interaction of killer cell immunoglobulin-like receptors with ligands present on leukemic cells [39].

### Other New Drugs

Studies have evaluated new agents such as clofarabine and newer formulations of older cytotoxic drugs. In 25 children with relapsed/refractory ALL treated with intravenous clofarabine, administered in combination with cyclophosphamide and etoposide (NCT00315705), 44% maintained remission over a median 67 wk [40]. Toxicity is high with clofarabine-based regimens, limiting regular use.

Liposomal formulations of conventional cytotoxic agents have been designed to enhance drug efficacy and/or lower drug-related toxicity. Pegylated liposomal doxorubicin is potentially less cardiotoxic but not more effective [41]. Erythrocyte encapsulated asparaginase (Graspa) has not been found to be more effective than pegylated asparaginase in a randomized trial [42].

## Precision Therapy

Precision therapy in oncology has traditionally targeted genomic alterations. Targeted therapy based on tumor molecular profiling did not, however, improve outcomes in a large, randomized phase 2 trial [43]. An alternative strategy is to use drug response profiling (DRP) with using a panel of drugs to identify suitable agents for patients with refractory disease, an approach referred to as functional precision oncology. This approach was tested prospectively in 76 patients with advanced hematological malignancies where outcomes were compared in patients receiving DRP-based therapy (n = 56) *vs.* physician-choice therapy (n = 20). At a median 23.9 mo, improved PFS (1.3-fold benefit) was observed in nearly half of patients (30 patients, 54%) who received DRP-based therapy [44]. DRP of 56 primary samples of pediatric patients with relapsed/refractory ALL identified lymphoblast sensitivity to bortezomib and venetoclax in a subset with poor treatment response, and concomitant 3-fold improvements in remission rates (from 28 to 85%) was observed when the drugs were added for these patients [45]. This strategy can be used further to identify potent synergistic drug combinations in high risk malignancies.

## Approach to Treatment and Supportive Care

The diagnosis of relapse is an intensely distressing experience for patients and families and requires an empathetic approach. In addition to the variables that influence risk-directed management of relapsed ALL (such as age, ALL lineage, genetics, timing & site of relapse), management plan also includes review of first presentation ALL treatment for information of drug-related toxicities (*e.g.,* asparaginase-associated hypersensitivity), drug exposure (*e.g.,* cumulative anthracycline dose) and radiation treatment. Invasive bacterial and fungal infections result in life-threatening toxicity and treatment-related mortality, especially during the induction treatment phase [7, 11]. The authors recommend antifungal infection prophylaxis with liposomal amphotericin B during the induction and intensification treatment phases of InPOG-ALL-R1. Patients treated with radiation therapy, either for extramedullary disease or as part of HSCT conditioning, are at risk of endocrine morbidities and second malignancies and require long-term monitoring.

## Conclusions

At authors’ center, initially many patients refused curative treatment mostly due to cost of treatment. No treatment, however, leads to painful death. For these patients, authors initiated low-cost chemotherapy to support the child for as long as possible. In authors’ experience, this is associated with a median survival of around 3 mo, requires blood product support but usually keeps the child out of hospital. Increasingly, more patients are now opting for curative therapy but cannot afford HSCT. In these high-risk patients, authors have found that by using intensive chemotherapy, it is possible to achieve a prolonged high quality progression-free survival [11]. With recent introduction of a blinatumomab donor program and drug response profiling, authors are now able to maintain remissions for longer durations, even in very high-risk patients.

Understanding the biology of relapse, development of newer therapies and approaches provide hope for improvement of survival in children with relapsed ALL. Concerted efforts are required to identify solutions that would be accessible globally.
